# Confinement-induced stabilization of the Rayleigh-Taylor instability and transition to the unconfined limit

**DOI:** 10.1126/sciadv.abd6605

**Published:** 2020-11-18

**Authors:** Samar Alqatari, Thomas E. Videbæk, Sidney R. Nagel, A. E. Hosoi, Irmgard Bischofberger

**Affiliations:** 1 Department of Physics and The James Franck and Enrico Fermi Institutes, University of Chicago, Chicago, IL 60637, USA.; 2 Department of Mechanical Engineering, Massachusetts Institute of Technology, Cambridge, MA 02139, USA.

## Abstract

The prevention of hydrodynamic instabilities can lead to important insights for understanding the instabilities’ underlying dynamics. The Rayleigh-Taylor instability that arises when a dense fluid sinks into and displaces a lighter one is particularly difficult to arrest. By preparing a density inversion between two miscible fluids inside the thin gap separating two flat plates, we create a clean initial stationary interface. Under these conditions, we find that the instability is suppressed below a critical plate spacing. With increasing spacing, the system transitions from the limit of stability where mass diffusion dominates over buoyant forces, through a regime where the gap sets the wavelength of the instability, to the unconfined regime governed by the competition between buoyancy and momentum diffusion. Our study, including experiment, simulation, and linear stability analysis, characterizes all three regimes of confinement and opens new routes for controlling mixing processes.

## Teaser

Sufficient confinement can completely suppress the Rayleigh-Taylor instability between two density-inverted miscible fluids.

## INTRODUCTION

The Rayleigh-Taylor instability arises when a dense fluid sinks and displaces a lighter one located below it (*1*). Of particular interest is the case of miscible fluids, where surface tension is negligible and interdiffusion of the fluids is important. This is relevant in diverse situations: In astrophysics, improved models of the instability in type Ia supernovae are needed to estimate the universe’s cosmological expansion (*2*, *3*). In nuclear engineering, Rayleigh-Taylor mixing occurs in inertial confinement fusion, preventing ignition (*4*–*6*). In geophysics, the Rayleigh-Taylor mechanism drives the formation of salt fingers (*7*, *8*).

**Fig. 1:**
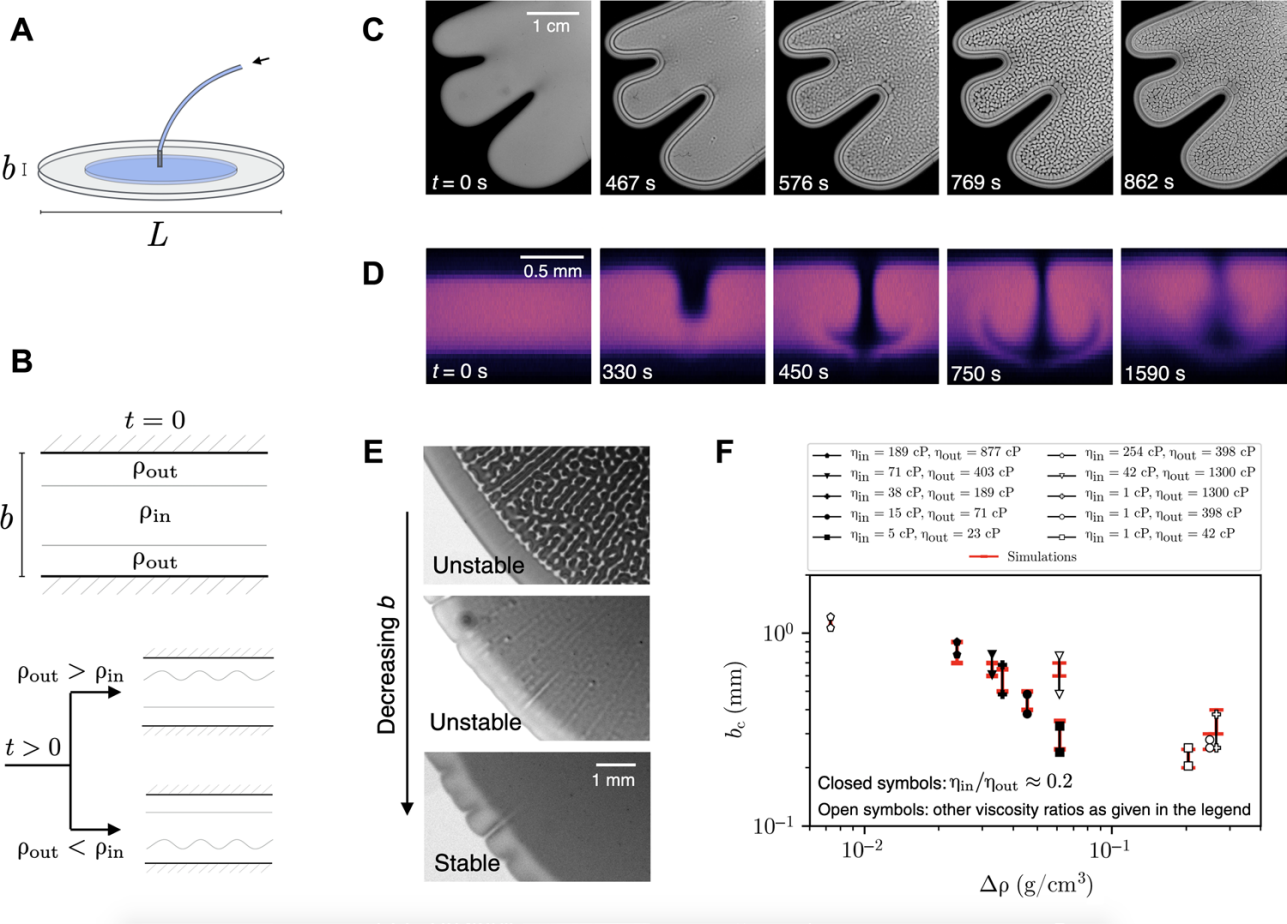
Confinement induces stabilization. (**A**) Hele-Shaw cell with gap thickness 0.05 < *b* < 1.2 mm and *b* ≪ *L*. (**B**) Density profile within a segment of the gap. Top: Initial density profile after injection of the inner fluid. Bottom: When the outer fluid is denser, the top interface becomes unstable; when the inner one is denser, the bottom interface becomes unstable. (**C**) Temporal evolution of the Rayleigh-Taylor instability, viewed from the bottom with *b* = 1.18 mm and fluids with η_out_ = 1150 cP, η_in_ = 32.4 cP, Δρ = 0.0069 g/cm^3^, and an effective interfluid diffusion coefficient *D* = 2.8 × 10^−6^ cm^2^/s. *t* = 0 is when injection ceases. (**D**) Time series of the instability viewed with a confocal microscope, for fluids with η_out_ = 610 cP, η_in_ = 101 cP, Δρ = 0.031 g/cm^3^, and *D* = 0.52 × 10^−6^ cm^2^/s. (**E**) Decreasing the gap thickness (*b*= 406, 330, and 241 μm) for a fixed set of fluids (η_out_ = 29 cP, η_in_ = 5.0 cP, Δρ = 0.070 g/cm^3^, and *D* = 2.92 × 10^−6^ cm^2^/s) leads to the disappearance of the instability at small enough plate spacings. (**F**) The critical plate spacing, *b*_c_, for pairs of miscible fluids versus the density difference Δρ. Black symbols denote experimental results, and red symbols are from simulations.

Creating a controlled unstable density-inverted situation is an experimental challenge but crucial in a field driven by theory (*9*–*13*) and simulations (*14*, *15*). Experiments usually involve rapid acceleration of the fluids in a drop tower, rotation, or barrier removal (*16*–*20*). The latter methods often introduce perturbations due to induced vorticity or viscous drag that can dominate instability growth. Here, we use a horizontal Hele-Shaw geometry, consisting of two parallel plates separated by a thin gap of size *b*, as illustrated in Fig. 1A. When one fluid is injected at low Reynolds number into the gap containing a second fluid with which it is miscible, a well-defined protruding “tongue” is formed (*21*–*23*) where a density inversion can occur.

This geometry allows us to study the Rayleigh-Taylor instability with clean initial conditions under confinement. This adds an unexpected feature: Below a critical plate spacing, *b*_c_, the Rayleigh-Taylor instability remarkably no longer occurs. This demonstrates the existence of an additional important length scale when nearby boundaries are present. While stabilization at small plate spacing has previously been reported in the Rayleigh-Taylor instability with flowing liquids (*24*), the instability in that case is dominated by the flow-induced stretching of the density profile (*25*). Those flows alter both the wavelength selection and the dynamics of the instability compared to the quiescent initial state investigated here.

Here, we analyze the competition between the destabilizing effect of buoyancy and the stabilizing effects of both momentum and mass diffusion. Duff *et al*. (*12*) included the effect of mass diffusion into the theory of the instability. Aside from the gap spacing *b*, there are two distinct length scales: one, λ_∞_, is associated with momentum diffusion and sets the most unstable wavelength for pattern growth in the unconfined limit; the other, *b*_c_, is associated with mass diffusion and sets the minimum plate spacing for unstable growth. We find that at intermediate scales, the gap spacing controls the most unstable wavelength, λ. By varying the confinement length scale, we can study the different regimes and tune the relative importance of mass and momentum diffusion.

## RESULTS

### Experiments

In our experiment, the protruding tongue of inner fluid spontaneously produces a stratification into three fluid layers with well-defined interfaces between them, as shown in Fig. 1B. When the outer fluid has a higher density than the inner one, ρ_out_ > ρ_in_, the upper interface is unstable; when ρ_in_ > ρ_out_, the lower interface is unstable. For our range of plate spacings, injection rates, and fluid viscosities used, the Reynolds number is small so that the fluids come to rest almost instantaneously, providing clean initial conditions. Cell-like structures and lines then appear, as shown in Fig. 1C. The alternating intensities represent the peaks and valleys of the unstable interface. We visualize the evolution of these structures within the gap by imaging the cell’s cross section using a confocal microscope. As the denser fluid on top sinks into the lighter fluid in the center, the encroaching fluid develops the characteristic mushroom shape of the Rayleigh-Taylor instability, as shown in Fig. 1D.

The instability is suppressed below a critical plate spacing, as shown in Fig. 1E for the same pair of fluids in cells of decreasing gap thickness, *b*; the patterns seen clearly at large plate spacing gradually fade until they disappear below a critical spacing, *b*_c_. Note that the patterns here form preferentially in the direction radial from the inlet. This is most pronounced at high viscosity ratio. We believe that this feature of the pattern is due to a slight radial dependence of the thickness of the inner fluid tongue, which adds a small slope to the interface as compared with a flatter interface at low viscosity ratios shown in Fig. 1C. This effect is robust and observed over the range of density differences Δρ = ∣ ρ_out_ − ρ_in_∣ that we can access. For a fixed viscosity ratio, η_in_/η_out_ ≈ 0.2, *b*_c_ decreases monotonically with Δρ, as shown in data with solid symbols in Fig. 1F. The open symbols show data for other values of η_in_/η_out_. We represent *b*_c_ as a bar denoting where the system transitions from stability to instability. The upper end of these bars indicates the smallest *b* where we observe instability and the lower end denotes the largest *b* where we do not see measurable perturbations. An advantage of using water-glycerol mixtures is that their fluid properties are well characterized (*26*–*28*). However, one cannot independently vary one parameter without also changing the others. This makes parts of parameter space inaccessible.

### Simulations

We take advantage of two-dimensional (2D) simulations to explore the parameters governing this stabilization systematically. We validate our simulations by replicating the three-layer geometry and complex fluid properties of our experimental setup. This results in excellent agreement with the experiments, as shown by the red symbols in Fig. 1F, where we determine the critical plate spacing *b*_c_ by incrementally decreasing *b* until the instability disappears. Our simulations thus accurately model the system and can be used to extract information about *b*_c_.

The experimental three-layer fluid profile of the Hele-Shaw cell, replicated in the simulations, creates a configuration with many relevant length scales, including the thickness of each layer. We therefore simplify the geometry to two fluid layers, each occupying half the gap. We use an average concentration-independent mass diffusivity, *D*, and fix the viscosity of the fluids such that η = η_H_ = η_L_, where η_H_ and η_L_ denote the viscosities of the higher and lower density fluids, respectively.

**Fig. 2:**
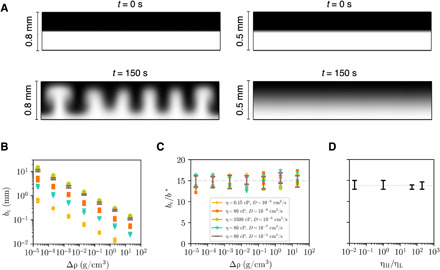
Control parameters for stabilization probed in a simplified system. (**A**) Initial and final states for two-layer 2D simulations at *b* = 0.8 mm (unstable) and *b* = 0.5 mm (stable). (**B**) The critical plate spacing *b*_c_ versus Δρ for a range of viscosities and diffusion coefficients. (**C**) Normalizing *b*_c_ with b* = (η*D*/*g*Δρ)^1/3^ collapses the data to *b*_c_/b* = 15.0 ± 0.9. (**D**) *b*_c_/b* as η_H_/η_L_ is varied while keeping 〈η〉 constant.

The simplified simulations again capture the transition to stability at a critical plate spacing, as shown in Fig. 2A. As shown in Fig. 2B, *b*_c_ increases with increasing either *D* or η, indicating that diffusion of mass and momentum act to stabilize the interface. By contrast, increasing Δρ leads to a decrease in *b*_c_, indicating that buoyancy forces *g*Δρ (where *g* is the Earth’s gravitational constant) drive the instability. We therefore normalize the critical plate spacing by a characteristic length formed only from *D*, η, and *g*Δρ

$$b^{*}\propto {\left(\frac{\eta D}{g\Delta \rho }\right)}^{1/3}$$ (1)

Over six decades of density difference, the data collapse with *b*_c_/b* = 15.0 ± 0.9, as shown in Fig. 2C. This corroborates the hypothesis that *b*_c_ is determined by a competition between buoyancy and the stabilizing effects of viscosity and mass diffusion.

Note that Eq. 1 is analogous to the Rayleigh number for the Rayleigh-Bénard instability (*29*) typically used to denote the balance between thermal diffusion and bouyancy in systems with a temperature gradient: Ra = Δρ*gl*^3^/η*D*_t_. Here, *l* is a characteristic length scale and *D*_t_ is the coefficient of thermal diffusion (*30*). In our system, mass diffusion, *D*, replaces the stabilizing role of thermal diffusion in the Rayleigh-Bénard instability. Chandrasekhar (*29*) determined the critical Rayleigh number for a bounded geometry as Ra_c_ = 1707.8, below which no convection cells appear. If we use our expression for b*, but using *D*_t_ instead of *D*, the critical length scale for the Rayleigh-Bénard problem is *l*/b* ≈ 12, which is similar to *b*_c_/b* = 15.0 ± 0.9 that we find for our instability. Although the system that we consider does not have continual driving required to sustain long-lived convective cells, the mechanisms responsible for the stability are similar in these two systems and it is reasonable that they have similar length scales governing the transition to stability.

To investigate the case where the fluids have different viscosities, we determine *b*_c_ as a function of the viscosity ratio, η_H_/η_L_, while holding the average viscosity, 〈η〉, constant. With b* ≡ (〈η〉*D*/*g*Δρ)^1/3^, the data again collapse to *b*_c_/b* = 13.8 ± 0.6, as shown in Fig. 2D. We conclude that the viscosity ratio does not substantially affect *b*_c_.

**Fig. 3:**
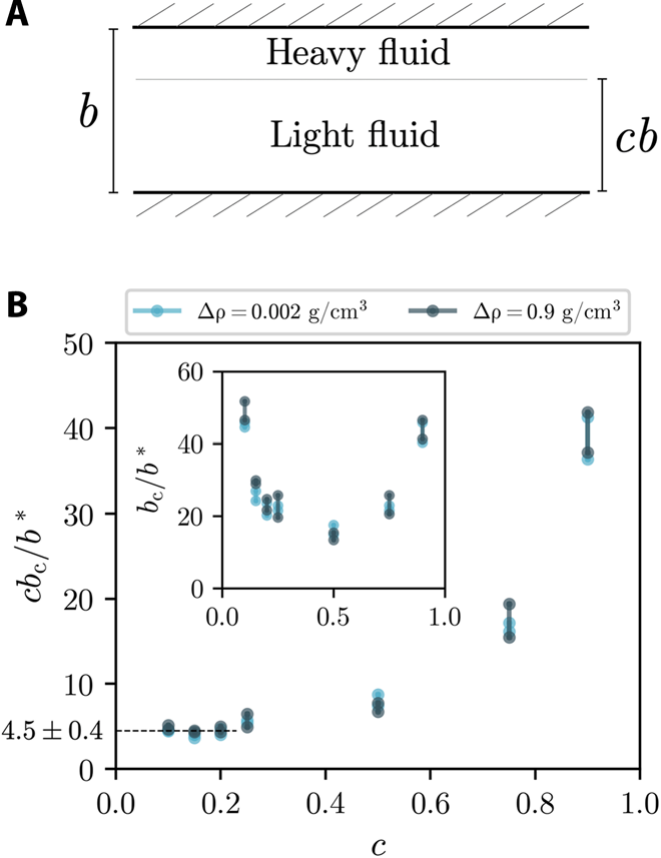
Relevant length scale for confinement. (**A**) Schematic of simulated interface located at height *cb* in the gap; 0 < *c* < 1. (**B**) Distance to the lower wall, *cb*_c_/b*, at the critical stability point versus *c*. At low *c*, *cb*_c_/b* = 4.5 ± 0.4, independent of Δρ. Inset: *b*_c_/b* increases as the interface approaches either boundary.

When located in the middle of the gap, the interface is equally sensitive to both boundaries. To determine the effect of each boundary separately in the simulations, we locate the interface at different heights from the bottom plate, *cb*, with 0 < *c* < 1, as shown in Fig. 3A. The inset of Fig. 3B shows that *cb*_c_/b* has a symmetric minimum at *c* = 0.5 independent of Δρ. As *c* approaches either 0 or 1, the confinement-induced stabilization increases, suggesting that, as one might expect, the thinner layer has a greater importance than the thicker one. As *cb* → 0, *cb*_c_/b* plateaus at 4.5 ± 0.4 (Fig. 3B). This is the value associated with only one boundary. As *c* increases, *cb*_c_/b* increases as the interface becomes more influenced by the upper boundary as well.

### Linear stability analysis

**Fig. 4:**
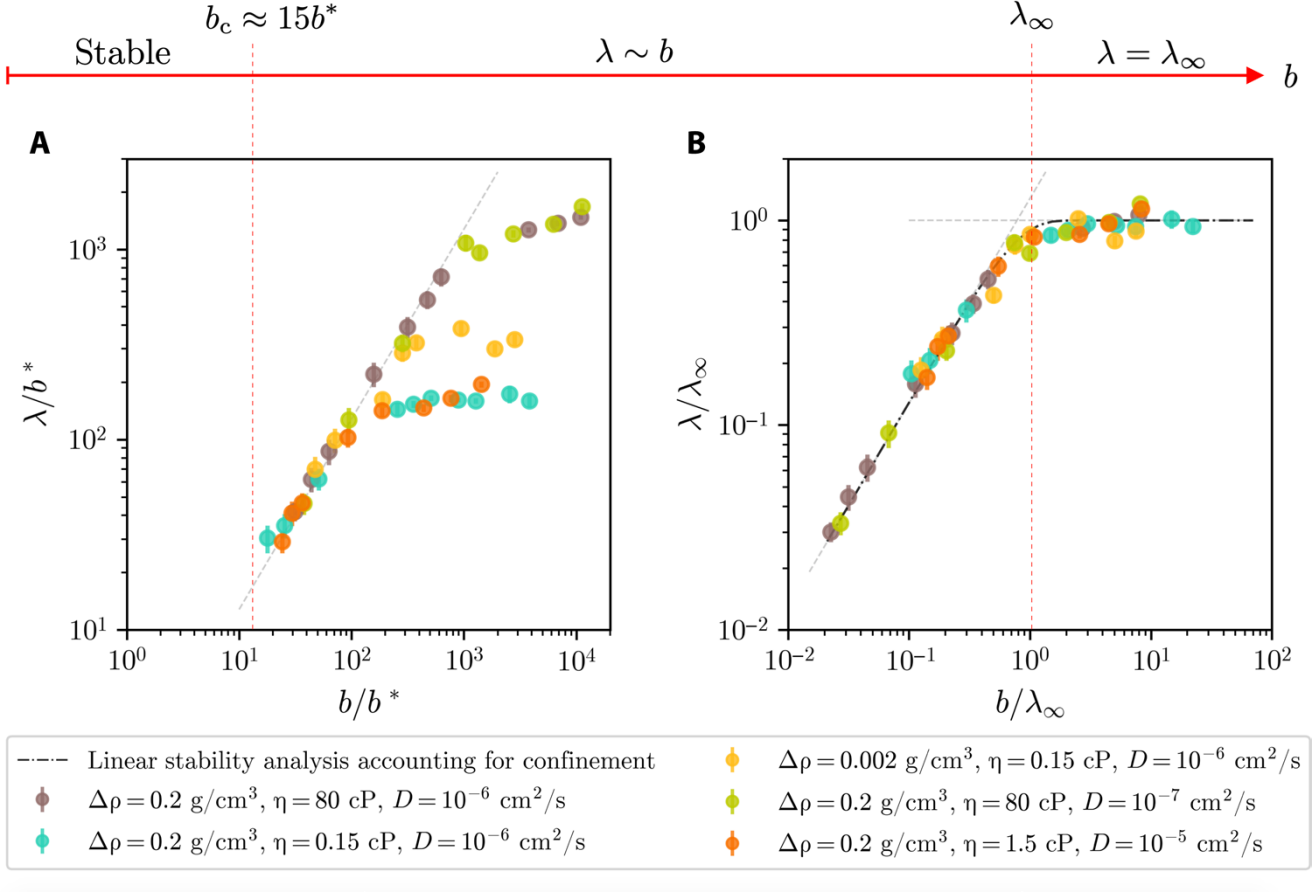
From stability to open space. (**A**) Dependence of λ on *b* for a range of fluid parameters (legend) from simulations. Linear stability analysis accounting for confinement predicts λ ≈ 1.28*b* for b* < *b* < λ_∞_ (gray dashed line). The red dashed line denotes the minimum *b* before stability at *b*/b* ≈ 15. Error bars represent the discretization error from the wavelength being an integer fraction of the channel width. (**B**) Normalization with λ_∞_ collapses all data. The transition to the unconfined limit occurs when *b* ≈ λ_∞_ (red dashed line). Results from the linear stability analysis are shown by the black dashed-dotted line.

To probe confinement effects above *b*_c_, we measure the spontaneously selected instability wavelength, λ, versus the plate spacing. Figure 4A shows λ/b* versus *b*/b*. The red dashed line indicates *b*_c_ below which no instability exists. Above that cutoff, λ initially grows linearly with *b* until it plateaus to a constant.

To understand this plateau at large *b*, we consider the linear stability analysis by Duff *et al*. (*12*) in the absence of confinement. The instability growth rate (in terms of the wave number *k* = 2π/λ) is

$$n=\sqrt{\mathrm{Agk}/\psi (A,t)+\nu^{2}k^{4}}-(\nu +D)k^{2}$$ (2)

where *A* = (ρ_1_ − ρ_2_)/(ρ_1_ + ρ_2_) is the Atwood number, ν ≡ (η_1_ + η_2_)/(ρ_1_ + ρ_2_) is the average kinematic viscosity, and ψ is a function of *A*, *k*, and the interface thickness $\delta =\sqrt{2\mathrm{Dt}}$. Initially, before the interface has significantly diffused, *k*δ ≪ 1 and ψ ≈ 1 for all Atwood numbers. For liquids, it is typical that ν ≫ *D*. In this limit, Eq. 2 yields the most unstable wavelength

$${\lambda_{\infty }\equiv \lambda |}_{\mathrm{dn}/\mathrm{dk}=0}\approx 4\pi {\left(\frac{\nu^{2}}{\mathrm{gA}}\right)}^{1/3}=2^{8/3}\pi {\left[\frac{\eta^{2}A}{g{\left(\Delta \rho \right)}^{2}}\right]}^{1/3}$$ (3)

where η = η_1_ = η_2_. λ_∞_ corresponds to the plateau of the measured wavelength, as shown in Fig. 4B where we normalize *b* and λ with λ_∞_. The onset of the plateau occurs when *b* ≈ λ_∞_. Thus, λ_∞_ sets the scale beyond which the instability no longer feels the confinement. We note that λ_∞_ is fairly insensitive to mass diffusion and governed only by the competition between momentum diffusion and buoyancy. In the unconfined limit, the instability growth rate is always large enough that the time scale for dynamics is shorter than for mass diffusion.

To account for the confinement-dominated intermediate regime, we add in additional boundary conditions to Chandrasekhar’s unconfined linear stability analysis (*9*, *31*) (see the Supplementary Materials). This modified theory, shown by the dashed-dotted line in Fig. 4B, is in excellent agreement with the data and yields λ ≈ 1.28*b* for *b* < λ_∞_. This analysis further shows that decreasing *b* leads to a decreasing growth rate; when *b* approaches *b*_c_, the time for growth becomes of the same magnitude as the characteristic time for diffusion. At small enough gaps, the interface between the fluids diffuses before the instability can grow by buoyant forces.

## DISCUSSION

Accounting for mass diffusion in the linear stability analysis introduces a second characteristic length that is not present when only viscosity is considered: a cutoff wavelength at which the growth rate approaches zero, λ_cutoff_ = λ∣_*n* = 0_

$$\lambda_{\text{cutoff}}\approx 2\pi {\left(\frac{2\nu D}{\mathrm{gA}}\right)}^{1/3}=2^{5/3}\pi {\left(\frac{\eta D}{g\Delta \rho }\right)}^{1/3}$$ (4)

λ_cutoff_, marking the transition between stable and unstable modes, is the smallest unstable wavelength allowed. λ_cutoff_ scales in the same way as b*. Our interpretation of *b*_c_ as the result of a competition between driving and stabilizing forces is thus consistent with the theoretical prediction of a cutoff wavelength in an unbounded, miscible system. Only by going to a confined system though does this length scale become accessible.

The Rayleigh-Taylor instability for miscible fluids in confinement is thus characterized by two independent lengths: (i) *b*_c_ sets a minimum length scale for the instability and is dominated by mass diffusion; (ii) λ_∞_, dominated by momentum diffusion, gives the scale beyond which the system no longer feels the confinement. In between, the wavelength is controlled by *b*. Varying the plate spacing allows one to approach one or the other limit systematically.

In conclusion, the presence of a wall close to a two-fluid interface decreases the most unstable wavelength and slows down the instability growth. Varying confinement could provide a novel means for controlling mixing in miscible fluids; it allows a transition between purely diffusion-dominated mixing to a much faster instability-dominated mixing. Such a transition is similar to the switch from laminar diffusion to convective mixing at a critical value of the Rayleigh number in the Rayleigh-Bénard instability (*24*, *30*). Stabilization from confinement might be exploited in systems where instabilities are detrimental, such as in inertial confinement fusion where instabilities can prevent ignition (*32*).

## MATERIALS AND METHODS

### Experimental

The glass plates used in our Hele-Shaw experiments are of diameter 28 cm and thickness 1.9 cm. A uniform gap between the plates is maintained using spacers of thickness *b* = [0.05,1.2] mm placed along the perimeter of the cell. We use miscible fluids composed of glycerol-water mixtures with viscosities between 0.932 and 1078 cP and densities between 0.997 and 1.258 g/cm^3^ at 23°C. Fluid viscosities and densities are measured using an SVM 3001 viscometer (Anton Paar). To differentiate the fluids, we dye one of them with Brilliant Blue G (Alfa Aesar). We first inject an outer fluid through a hole in the center of one of the plates and then inject another inner fluid. We image the patterns from the bottom of the cell with a Prosilica GX3300 camera at frame rates ranging from 0.01 to 14 frames/s.

We use confocal microscopy (Caliber I.D. RS-G4 confocal microscope) to visualize the structure within the gap inside a rectangular Hele-Shaw cell of size 2.5 cm by 7.5 cm and plate spacing *b* = 1.14 mm. The inner fluid is dyed with Rhodamine B (Sigma-Aldrich) at a concentration of 2 μM. As a single stack with 31 *z* slices takes about 30 s to capture, we use higher-viscosity fluids to slow down the dynamics, so that the instability takes 6 min to appear with full development occurring after 15 min. Our acquisition rate is then fast compared to the dynamics that we wish to capture.

### Numerical

We complement the experiments with 2D numerical simulations of the cross section of the Hele-Shaw cell. We model the two miscible fluids as a mixture with mass concentration ω*_i_* = [0, 1]. Using COMSOL, we solve the Stokes equations for creeping flow coupled with Fick’s law for diffusion

$$\begin{array}{c} \rho \nabla \cdot u=0\\ \nabla \cdot [-pI+\eta (\nabla u+{\left(\nabla u\right)}^{T})]+\rho g=0\\ \rho \frac{\partial \omega_{i}}{\partial t}+\nabla \cdot j_{i}+\rho (u\cdot \nabla )\omega_{i}=0 \end{array}$$ (5)

where

$$\begin{array}{c} j_{i}=-\rho D\nabla \omega_{i}-{\rho \omega }_{i}D\frac{\nabla M_{n}}{M_{n}}+{\rho \omega }_{i}\underset{k}{\Sigma }\frac{M_{i}}{M_{n}}D\nabla x_{k}\\ M_{n}={\left(\underset{i}{\Sigma }\frac{\omega_{i}}{M_{i}}\right)}^{-1} \end{array}$$ (6)

ρ is the mixture density, **u** is the flow velocity, *p* is the pressure, η is the mixture dynamic viscosity, *D* is the mixture diffusion coefficient, *M_i_* is the molar mass of species *i*, and *x_k_* is the molar fraction of species *k*. The cross section of the Hele-Shaw cell is a rectangular domain of height *b* and width *L* = [10,16]*b*, large enough for the dynamics to be independent of the domain size. We impose no-slip and no-flux boundary conditions at the top and bottom of the domain and periodic boundary conditions on the left and right. We follow (*26*) in modeling ρ as a linear interpolation between the two fluid densities. The fluid interface is perturbed using a smooth random function and smoothed with an error function. The initial interface thickness is δ ≤ 0.01*b* and the perturbation amplitude is *a* ≲ δ. The mesh size Δ*x* is a fraction of *a*, uniformly spaced around the interface where dynamics are pertinent. With time, the unstable interface chooses a wavelength independent of *L*.

We validate the model by comparing the simulation results to experiments performed under identical conditions, using the same fluid parameters and replicating the three-layer fluid profile within the gap. We use empirical formulas for both the interfluid diffusivity (*27*) and the viscosity (*28*) of glycerol-water mixtures as a function of the local mixture concentration, ω*_i_*. Such care needs to be taken to achieve agreement between the simulations and the experiments.

We then run another set of simulations, simplifying the geometry to two fluid layers with a single interface so that we can probe the effect of the interface location systematically. In addition, we simplify the fluid parameters, replacing the concentration-dependent diffusivity and viscosity with a constant average diffusivity, *D*, and a viscosity that interpolates linearly with the local concentration, ω*_i_*. The empirical relations are asymmetric with respect to ω*_i_*, which leads to a drift in the location of the fluid interface as the system diffuses. The simplification of the fluid parameters is necessary to decouple the effect of the interface location from that of the fluid properties.

## Supplementary Material

http://advances.sciencemag.org/cgi/content/full/6/47/eabd6605/DC1

Adobe PDF - abd6605_SM.pdf

Confinement-induced stabilization of the Rayleigh-Taylor instability and transition to the unconfined limit

## SUPPLEMENTARY MATERIALS

Supplementary material for this article is available at http://advances.sciencemag.org/cgi/content/full/6/47/eabd6605/DC1
