# Supplementary material for: Confinement-induced stabilization of the Rayleigh-Taylor instability and transition to the unconfined limit
Source: Sci Adv. 2020 Nov 18;6(47):eabd6605. doi: 10.1126/sciadv.abd6605 (PMC7673797; doi:10.1126/sciadv.abd6605)
Supplement: http://advances.sciencemag.org/cgi/content/full/6/47/eabd6605/DC1 [file supp_6_47_eabd6605__index.html]

Science Advances | Science AdvancesAAASSearchScience AdvancesMenu

## Supplementary Materials

# Confinement-induced stabilization of the Rayleigh-Taylor instability and transition to the unconfined limit

Samar Alqatari, Thomas E. Videbæk, Sidney R. Nagel, A. E. Hosoi, Irmgard Bischofberger

Download Supplement

**This PDF file includes:**

- Linear stability analysis with boundaries
- Figs. S1 and S2

**Files in this Data Supplement:**

- Adobe PDF - abd6605\_SM.pdf
