# Supplementary material for: Confinement-induced stabilization of the Rayleigh-Taylor instability and transition to the unconfined limit
Source: Sci Adv. 2020 Nov 18;6(47):eabd6605. doi: 10.1126/sciadv.abd6605 (PMC7673797; doi:10.1126/sciadv.abd6605)
Supplement: http://advances.sciencemag.org/cgi/content/full/6/47/eabd6605/DC1 [file supp_6_47_eabd6605__1.pdf]

Science Advances
AAAS

advances.sciencemag.org/cgi/content/full/6/47/eabd6605/DC1

Supplementary Materials for

Confinement-induced stabilization of the Rayleigh-Taylor instability and transition to the unconfined limit

Samar Alqatari*, Thomas E. Videbæk, Sidney R. Nagel, A. E. Hosoi, Irmgard Bischofberger

*Corresponding author. Email: samarq@uchicago.edu

Published 18 November 2020, *Sci. Adv.* **6**, eabd6605 (2020)
DOI: 10.1126/sciadv.abd6605

This PDF file includes:

Linear stability analysis with boundaries
Figs. S1 and S2

Linear-Stability Analysis with Boundaries

Single boundary

To probe how the most unstable wavelength is affected by confinement we incorporate two walls into the linear-stability analysis of Chandrasekhar (*9*), as illustrated in Fig. S1A. Two fluids with densities ρ_1 and ρ_2, and dynamic viscosities η_1 and η_2 form an interface located between two walls a distance b apart at a location cb, with $0 < c < 1$. To begin we set $c = 0.5$ to match the majority of the simulations reported in the manuscript. Note that we do not include the effect of mass diffusion in this analysis. We here show the dispersion relation and the wavelengths that result from this linear-stability analysis; details of the calculation of the dispersion relation are provided in the next section.

For now we restrict ourselves to the case of $\nu_1 = \nu_2$, where $\nu = \eta/\rho$. Fig. S1B shows the dispersion relation for varying b, where n is the instability growth rate and k is the wavenumber of a disturbance at the fluid-fluid interface. The change in color from black to red denotes a decrease in b. The location of the maximum growth rate, where $\partial_k n = 0$, indicates the most unstable wavenumber that will be selected by the system. The magnitude of the growth rate decreases and the most unstable wavenumber increases as we make b smaller. The most

unstable wavelength, $\lambda = 2\pi/k$, increases linearly with the plate spacing at small b and plateaus at larger b, as shown in Fig. S1C. In the linear regime, $\lambda \approx 1.28b$.

We compare λ to the most unstable wavelength λ_∞ selected when there is no boundary (*9*). In this unconfined case, for $\nu_1 = \nu_2$, the dispersion relation has the approximate form (*33, 34*)

$$n = \sqrt{gAk + \nu^2 k^4} - \nu k^2 \tag{1}$$

where $A = (\rho_2 - \rho_1)/(\rho_1 + \rho_2)$ is the Atwood number. This yields the most unstable wavelength

$$\lambda_\infty = 4\pi \left(\frac{\nu^2}{gA} \right)^{1/3}. \tag{2}$$

We use λ_∞ to rescale both λ and b in Fig. S1C. The plateau wavelength at large b is precisely λ_∞; the transition from the linear regime to open space limit is reached when $1.28b \approx \lambda_\infty$.

By varying the value of c we probe how the proximity of the interface to either boundary affects the wavelength. In exploring this we restrict ourselves to the confined regime, $b < \lambda_\infty$. The wavelength exhibits a maximum at $c = 0.5$ and decreases linearly as c approaches either 0 or 1, as shown in Fig. S1D. These two limits correspond to the limit where only one boundary matters and the interface does not feel the effect of the second wall. In this limit $\lambda \approx 3.7cb$, where cb is the distance to the nearest boundary.

As b decreases, the growth rate for the most unstable mode also decreases, as seen in Fig. S1B. The timescale for the growth of the instability thus increases and eventually becomes comparable to the diffusive timescale at small enough b. Comparing these two timescales allows us to estimate the critical plate spacing b_c below which the instability no longer occurs. We note that the growth rate for the full theory is always bounded by the approximate form from Hide (*33*), seen in Eqn. (1). At large k beyond the most unstable wavenumber, the growth rate is bounded by

$$n = \frac{1}{2} \frac{gA}{\nu} k^{-1}. \tag{3}$$

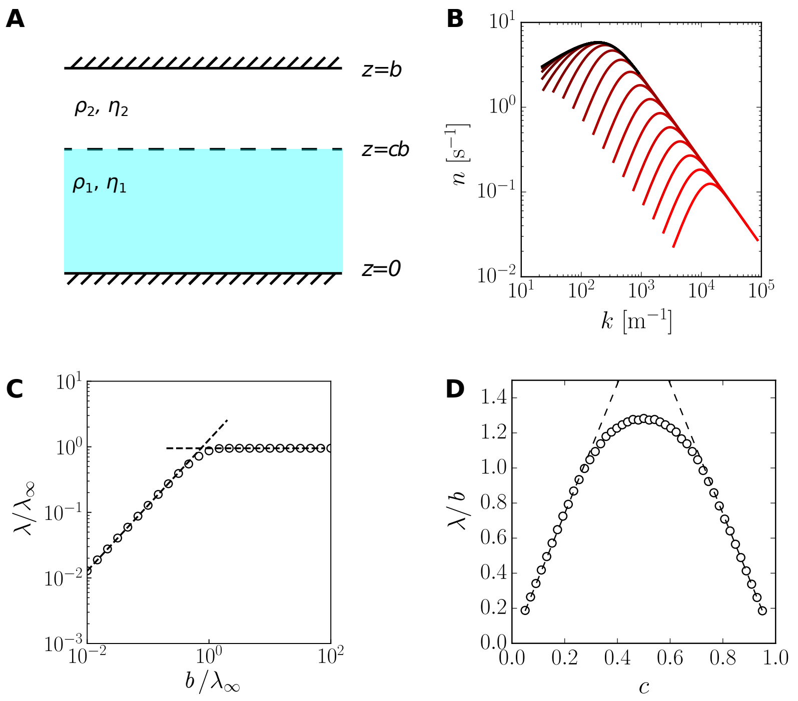

Figure S1: **Effect of gap separation on instability growth and wavelength.** **(A)** Schematic of two boundary geometry. The walls are a distance b apart with the interface located at cb; $0 < c < 1$. **(B)** Dispersion relations for $c = 0.5$ and $b \in [3.5 \cdot 10^{-4}, 3.5]$ m. Black curves correspond to large b and the curves become increasingly red as b decreases. **(C)** The most unstable wavelength, λ, as a function of b. Both axes have been scaled by the wavelength selected when no boundary is present, λ_∞. The sloped dashed line is $\lambda = 1.28b$, the horizontal dashed line is at $\lambda/\lambda_\infty = 1$. **(D)** The dependence of λ/b as a function of the interface location, c. The dashed lines have a slope of ± 3.7 corresponding to the limit of λ/b when one of the boundaries is infinitely far away. (**B-D**) All figures use fluid parameters $\eta_1 = 100$ cP, $\eta_2 = 110$ cP, $\rho_1 = 1$ g/cm^3, $\rho_2 = 1.1$ g/cm^3.

Since the most unstable wavelength is $\lambda \approx 1.28b$, we can estimate the growth rate n_c for this most unstable mode to be

$$n_c \approx \frac{1.28}{4\pi}\frac{gAb}{\nu}. \tag{4}$$

The characteristic timescale for diffusion is $b^2/4D$. By equating these two timescales we can estimate at what value of b they become comparable; this occurs when

$$b^3 = \frac{16\pi}{1.28}\frac{\nu D}{gA} = \frac{32\pi}{1.28}\frac{\eta D}{g\Delta\rho}. \tag{5}$$

If we interpret this length scale as the critical gap, then $b_c \approx 4.3b^*$, where $b^* = (\eta D/g\Delta\rho)^{1/3}$. This value is of the same order as the limiting case of $b_c \approx 15b^*$ shown in the main text. To reach a quantitative match, mass diffusion must be included into the linear-stability analysis.

Derivation of the dispersion relation

Here we show how to incorporate boundaries into the linear-stability analysis for the Rayleigh-Taylor instability. To reduce the amount of algebra and clearly identify the effect of incorporating a boundary we first show, in detail, the derivation for a single wall. This is followed by stating the result for two walls, adapting the same procedure as for one wall.

Dispersion matrix derivation: single boundary

To get the dispersion matrix we closely follow the work of Chandrasekhar (*9*) and Zetina et al. (*31*). We have the linearized Navier-Stokes equation:

$$(1 - \frac{\mu}{\rho n}(\partial_z^2 - k^2))(\partial_z^2 - k^2)w = 0 \tag{6}$$

where w is the z-component of the velocity field. The solutions of this equation are of the form:

$$w = \sum_{\pm} A_{\pm}e^{\pm kz} + B_{\pm}e^{\pm qz} \tag{7}$$

where $q^2 = k^2 + n/\nu$.

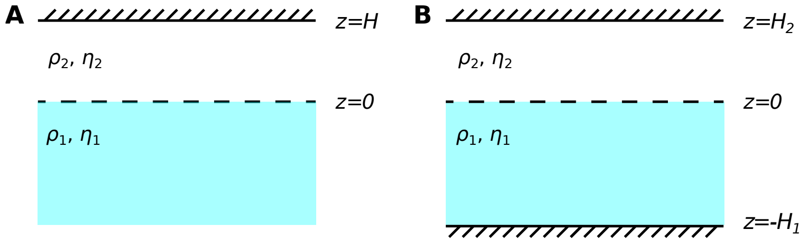

Figure S2: **Wall configurations considered in theoretical calculations.** **(A)** Schematic for single boundary geometry. The fluid located closer to the wall is fluid 2 and the wall is at a distance H away from the fluid-fluid interface. **(B)** Schematic for two boundary geometry. The upper and lower walls are distances H_2 and H_1 away from the fluid-fluid interface respectively.

The problem we address here is different from that in reference (*9*), in that we have a boundary located close to the unstable interface. The wall is at a distance H from the free boundary located at $z = 0$, see Fig. S2A. There are a few boundary conditions we need to satisfy. The first is that on any wall we require no-slip for the velocity field. This gives us two boundary conditions, $w = 0$ and $\partial_z w = 0$. We require this at $z = H$ and $z = -\infty$. From these boundary conditions we have that

$$w_1 = A_1 e^{kz} + B_1 e^{q_1 z} \tag{8}$$

$$w_2 = A_2 e^{-kz} + B_2 e^{-q_2 z} + C_2 e^{kz} + D_2 e^{q_2 z} \tag{9}$$

with index 1 and 2 referring to the lower and upper fluids. Using the boundary conditions on $z = H$ we get

$$A_2 e^{-kH} + C_2 e^{kH} + B_2 e^{-q_2 H} + D_2 e^{q_2 H} = 0 \tag{10}$$

$$-kA_2 e^{-kH} + kC_2 e^{kH} - q_2 B_2 e^{-q_2 H} + q_2 D_2 e^{q_2 H} = 0. \tag{11}$$

By multiplying equation (12) by k or q_2 and subtracting equation (13) we solve for C_2 and D_2

in terms of A_2 and B_2. This gives

$$C_2 = \frac{k+q_2}{k-q_2} e^{-2kH} A_2 + \frac{2q_2}{k-q_2} e^{-q_2 H - kH} B_2 \equiv \beta_1 A_2 + \beta_2 B_2 \tag{12}$$

$$D_2 = \frac{-2k}{k-q_2} e^{-q_2 H - kH} A_2 - \frac{k+q_2}{k-q_2} e^{-2q_2 H} B_2 \equiv \beta_3 A_2 + \beta_4 B_2 \tag{13}$$

where the β's are introduced for brevity.

To solve for the dispersion matrix we consider the boundary conditions on the unstable fluid-fluid interface. From Chandrasekhar (*9*) we have the following boundary conditions at $z = 0$:

$$w_1 = w_2 \tag{14}$$

$$\partial_z w_1 = \partial_z w_2 \tag{15}$$

$$\mu_1(\partial_z^2 + k^2) w_1 = \mu_2(\partial_z^2 + k^2) w_2 \tag{16}$$

$$\{[\rho_2 - \frac{\mu_2}{n}(\partial_z^2 - k^2)]\partial_z w_2\}_{z=0} - \{[\rho_1 - \frac{\mu_1}{n}(\partial_z^2 - k^2)]\partial_z w_1\}_{z=0}$$
$$= -\frac{gk^2}{n^2}(\rho_2 - \rho_1)[w]_0 - \frac{2k^2}{n}(\mu_2 - \mu_1)[\partial_z w]_0 \tag{17}$$

where $[w]_0$ and $[\partial_z w]_0$ are the common value at the interface (for w this could be the average value of w at the interface; but any choice of w_1, w_2, or the average are consistent). These four boundary conditions correspond to continuity of velocity, normal stress, tangential stress, and the kinematic boundary condition respectively.

By using the ansatz solutions for w_1 and w_2 and the substitutions for C_2 and D_2 we get the

following equations from each boundary condition:

$$A_1 + B_1 = A_2(1 + \beta_1 + \beta_3) + B_2(1 + \beta_2 + \beta_4) \tag{18}$$

$$kA_1 + q_1 B_1 = A_2(-k + k\beta_1 + q_2\beta_3) + B_2(-q_2 + k\beta_2 + q_2\beta_4) \tag{19}$$

$$\begin{aligned} A_1(2\mu_1 k^2) + B_1(\mu_1(q_1^2 + k^2)) = A_2(\mu_2(2k^2 + 2k^2\beta_1 + (q_2^2 + k^2\beta_3)) \\ +B_2(\mu_2((q_2^2 + k^2) + 2k^2\beta_2 + (q_2^2 + k^2)\beta_4)) \end{aligned} \tag{20}$$

$$\begin{aligned} A_1(\rho_1 - \frac{gk}{2n^2}(\rho_1 - \rho_2) - \frac{k^2}{n}(\mu_1 - \mu_2)) + B_1(-\frac{gk}{2n^2}(\rho_1 - \rho_2) - \frac{k^2}{n}(\mu_1 - \mu_2)\frac{q_1}{k}) \\ = A_2(\rho_2(1 - \beta_1) + \frac{gk}{2n^2}(\rho_1 - \rho_2)(1 + \beta_1 + \beta_3) + \frac{k^2}{n}(\mu_1 - \mu_2)(-1 + \beta_1 + \frac{q_2}{k}\beta_3)) \\ B_2(-\rho_2\beta_2 + \frac{gk}{2n^2}(\rho_1 - \rho_2)(1 + \beta_2 + \beta_4) + \frac{k^2}{n}(\mu_1 - \mu_2)(-\frac{q_2}{k} + \beta_2 + \frac{q_2}{k}\beta_4)). \end{aligned} \tag{21}$$

We define the variables:

$$\alpha_i = \rho_i/(\rho_1 + \rho_2) \tag{22}$$

$$R = \frac{gk}{2n^2}\frac{\rho_1 - \rho_2}{\rho_1 + \rho_2} \tag{23}$$

$$C = \frac{k^2}{n}\frac{\mu_1 - \mu_2}{\rho_1 + \rho_2}. \tag{24}$$

Using Eqns. (20) - (23) we look for non-trivial $A_1,\ B_1,\ A_2,\ B_2$ that satisfy them. We express these equations in matrix form such that

$$\mathbf{M} \cdot \mathbf{C} = 0,\ \mathbf{C} = \begin{bmatrix} A_1 \\ B_1 \\ A_2 \\ B_2 \end{bmatrix} \tag{25}$$

where $\mathbf{M}$ is

$$\mathbf{M} = \begin{vmatrix} 1 & 1 & -(1 + \beta_1 + \beta_3) & -(1 + \beta_2 + \beta_4) \\ k & q_1 & k(1 - \beta_1 - \frac{q_2}{k}\beta_3) & q_2(1 - \frac{k}{q_2}\beta_2 - \beta_4) \\ 2\mu_1 k^2 & \mu_1(q_1^2 + k^2) & \begin{matrix} -\mu_2(2k^2(1 + \beta_1) \\ +(q_2^2 + k^2)\beta_3) \end{matrix} & \begin{matrix} -\mu_2((q_2^2 + k^2)(1 + \beta_4) \\ +2k^2\beta_2) \end{matrix} \\ -R - C - \alpha_1 & -R - \frac{q_1}{k}C & \begin{matrix} -\alpha_2(1 - \beta_1) - R(1 + \beta_1 + \beta_3) \\ +C(1 - \beta_1 - \frac{q_2}{k}\beta_3) \end{matrix} & \begin{matrix} \alpha_2\beta_2 - R(1 + \beta_2 + \beta_4) \\ +C(\frac{q_2}{k} - \beta_2 - \frac{q_2}{k}\beta_4) \end{matrix} \end{vmatrix} \tag{26}$$

To ensure that the solution for $\mathbf{C}$ is non-trivial we need to find an $\mathbf{M}$ satisfying $\det(\mathbf{M}) = 0$. Calculating this determinant yields the dispersion relation that relates k and n. The calculations of the dispersion relation are done numerically.

Dispersion matrix derivation: two boundaries

We follow the same process for calculating the matrix $\mathbf{M}$ for the case of two boundaries, where the velocity for w_1 now has additional terms. We define the height above the interface as H_2 and the distance below the interface as H_1, as shown in Fig. S2B, and get the matrix

$$\begin{vmatrix} 1+\gamma_1+\gamma_3 & 1+\gamma_2+\gamma_4 & -(1+\beta_1+\beta_3) & -(1+\beta_2+\beta_4) \\ k(1-\gamma_1-\frac{q_1}{k}\gamma_3) & q_1(1-\frac{k}{q_1}\gamma_2-\gamma_4) & k(1-\beta_1-\frac{q_2}{k}\beta_3) & q_2(1-\frac{k}{q_2}\beta_2-\beta_4) \\ \begin{matrix}\mu_1(2k^2(1+\gamma_1) \\ +(q_1^2+k^2)\gamma_3)\end{matrix} & \begin{matrix}\mu_1((q_1^2+k^2)(1+\gamma_4) \\ +2k^2\gamma_2)\end{matrix} & \begin{matrix}-\mu_2(2k^2(1+\beta_1) \\ +(q_2^2+k^2)\beta_3)\end{matrix} & \begin{matrix}-\mu_2((q_2^2+k^2)(1+\beta_4) \\ +2k^2\beta_2)\end{matrix} \\ \begin{matrix}-\alpha_1(1-\gamma_1) \\ -R(1+\gamma_1+\gamma_3) \\ -C(1-\gamma_1-\frac{q_1}{k}\gamma_3)\end{matrix} & \begin{matrix}\alpha_1\gamma_2 \\ -R(1+\gamma_2+\gamma_4) \\ -C(\frac{q_1}{k}-\gamma_2-\frac{q_1}{k}\gamma_4)\end{matrix} & \begin{matrix}-\alpha_2(1-\beta_1) \\ -R(1+\beta_1+\beta_3) \\ +C(1-\beta_1-\frac{q_2}{k}\beta_3)\end{matrix} & \begin{matrix}\alpha_2\beta_2 \\ -R(1+\beta_2+\beta_4) \\ +C(\frac{q_2}{k}-\beta_2-\frac{q_2}{k}\beta_4)\end{matrix} \end{vmatrix} \tag{27}$$

with $H \to H_2$ in the β terms and the following definitions for the γ terms (note that they are the same as the β terms but with indices switched from 2 to 1 for the fluid identification)

$$\gamma_1 = \frac{k+q_1}{k-q_1}e^{-2kH_1} \tag{28}$$

$$\gamma_2 = \frac{2q_1}{k-q_1}e^{-q_1H_1-kH_1} \tag{29}$$

$$\gamma_3 = -\frac{2k}{k-q_1}e^{-q_1H_1-kH_1} \tag{30}$$

$$\gamma_4 = -\frac{k+q_1}{k-q_1}e^{-2q_1H_1}. \tag{31}$$

REFERENCES AND NOTES

1. G. I. Taylor, The instability of liquid surfaces when accelerated in a direction perpendicular to their planes I. *Proc. R. Soc. Lond. A* **201**, 192 (1950).
2. A. Burrows, Supernova explosions in the universe. *Nature* **403**, 727–733 (2000).
3. W. H. Cabot, A. W. Cook, Reynolds number effects on Rayleigh-Taylor instability with possible implications for type Ia supernovae. *Nat. Phys.* **2**, 562–568 (2006).
4. J. D. Kilkenny, S. Glendinning, S. W. Haan, B. Hammel, J. Lindl, D. Munro, B. Remington, S. Weber, J. Knauer, C. P. Verdon, A review of the ablative stabilization of the Rayleigh-Taylor instability in regimes relevant to inertial confinement fusion. *Phys. Plasmas* **1**, 1379–1389 (1994).
5. H.-S. Park, O. A. Hurricane, D. A. Callahan, D. T. Casey, E. L. Dewald, T. R. Dittrich, T. Döppner, D. E. Hinkel, L. F. B. Hopkins, S. Le Pape, T. Ma, P. K. Patel, B. A. Remington, H. F. Robey, J. D. Salmonson, J. L. Kline, High-adiabat high-foot inertial confinement fusion implosion experiments on the National Ignition Facility. *Phys. Rev. Lett.* **112**, 055001 (2014).
6. L. F. Wang, W. H. Ye, J. F. Wu, J. Liu, W. Y. Zhang, X. T. He, A scheme for reducing deceleration-phase Rayleigh-Taylor growth in inertial confinement fusion implosions. *Phys. Plasmas* **23**, 052713 (2016).
7. P. F. Linden, On the structure of salt fingers. *Deep Sea Res.* **20**, 325–340 (1973).
8. S. Zaleski, P. Julien, Numerical simulation of Rayleigh-Taylor instability for single and multiple salt diapirs. *Tectonophysics* **206**, 55–69 (1992).
9. S. Chandrasekhar, The character of the equilibrium of an incompressible heavy viscous fluid of variable density. *Math. Proc. Cambridge* **51**, 162–178 (1955).
10. S. I. Abarzhi, Review of theoretical modelling approaches of Rayleigh-Taylor instabilities and turbulent mixing. *Philos. T. Roy. Soc. A* **368**, 1809–1828 (2010).
11. H. J. Kull, Theory of the Rayleigh-Taylor instability. *Phys. Rep.* **206**, 197–325 (1991).
12. R. E. Duff, F. H. Harlow, C. W. Hirt, Effects of diffusion on interface instability between gases. *Phys. Fluids* **5**, 417 (1962).
13. D. D. Joseph, Fluid dynamics of two miscible liquids with diffusion and gradient stresses. *Eur. J. Mech. B Fluid* **6**, 565–596 (1990).
14. J. Fernandez, P. Kurowski, P. Petitjeans, E. Meiburg, Density-driven unstable flows of miscible fluids in a Hele-Shaw cell. *J. Fluid Mech.* **451**, 239–260 (2002).

15. M. A. Gallis, T. P. Koehler, J. R. Torczynski, S. J. Plimpton, Direct simulation Monte Carlo investigation of the Rayleigh-Taylor instability. *Phys. Rev. Fluids* **1**, 043403 (2016).
16. M. S. Roberts, J. W. Jacobs, The effects of forced small-wavelength, finite-bandwidth initial perturbations and miscibility on the turbulent Rayleigh-Taylor instability. *J. Fluid Mech.* **787**, 50–83 (2016).
17. J. T. Horne, A. G. W. Lawrie, Aspect-ratio-constrained Rayleigh-Taylor instability. *Physica D* **406**, 132442 (2020).
18. M. M. Scase, K. A. Baldwin, R. J. Hill, Magnetically induced rotating Rayleigh-Taylor instability. *J. Vis. Exp.* **121**, e55088 (2017).
19. G. Pacitto, C. Flament, J.-C. Bacri, C. M. Widom, Rayleigh-Taylor instability with magnetic fluids: Experiment and theory. *Phys. Rev. E* **62**, 7941–7948 (2000).
20. M. J. Andrews, S. B. Dalziel, Small Atwood number Rayleigh-Taylor experiments. *Philos. Trans. R. Soc. A.* **368**, 1663–1679 (2010).
21. E. Lajeunesse, J. Martin, N. Rakotomalala, D. Salin, Y. C. Yortos, Miscible displacement in a Hele-Shaw cell at high rates. *J. Fluid Mech.* **398**, 299–319 (1999).
22. I. Bischofberger, R. Ramachandran, S. R. Nagel, Fingering versus stability in the limit of zero interfacial tension. *Nat. Commun.* **5**, 5265 (2014).
23. T. E. Videbæk, S. R. Nagel, Diffusion-driven transition between two regimes of viscous fingering. *Phys. Rev. Fluids* **4**, 033902 (2019).
24. F. Haudin, L. A. Riolfo, B. Knaepen, G. M. Homsy, A. de Wit, Experimental study of a buoyancy-driven instability of a miscible horizontal displacement in a Hele-Shaw cell. *Phys. Fluids* **26**, 044102 (2014).
25. L. Talon, N. Goyal, E. Meiburg, Variable density and viscosity, miscible displacements in horizontal Hele-Shaw cells. Part 1. Linear stability analysis. *J. Fluid Mech.* **721**, 268–294 (2013).
26. Glycerine Producers' Association, *Physical Properties of Glycerine and Its Solutions* (Glycerine Producers Association, 1963).
27. G. D'Errico, O. Ortona, F. Capuano, V. Vitagliano, Data Diffusion coefficients for the binary system glycerol+ water at 25 C. A velocity correlation study. *J. Chem. Eng. Data* **49**, 1665–1670 (2004).

28. N.-S. Cheng, Formula for the viscosity of a glycerol-water mixture. *Ind. Eng. Chem. Res.* **47**, 3285–3288 (2008).
29. S. Chandrasekhar, *Hydrodynamic and Hydromagnetic Stability* (Oxford Univ. Press, 1961).
30. C. Normand, Y. Pomeau, M. G. Velarde, Convective instability: A physicists approach. *Rev. Mod. Phys.* **49**, 581 (1977).
31. S. Zetina, F. A. Godínez, R. Zenit, A hydrodynamic instability is used to create aesthetically appealing patterns in painting. *PLOS ONE* **10**, e0126135 (2015)
32. J. P. Sauppe, S. Palaniyappan, B. J. Tobias, J. L. Kline, K. A. Flippo, O. L. Landen, D. Shvarts, S. H. Batha, P. A. Bradley, E. N. Loomis, N. N. Vazirani, C. F. Kawaguchi, L. Kot, D. W. Schmidt, T. H. Day, A. B. Zylstra, E. Malka, Demonstration of scale-invariant Rayleigh-Taylor instability growth in laser-driven cylindrical implosion Experiments. *Phys. Rev. Lett.* **124**, 185003 (2020).
33. R. Hide, The character of the equilibrium of an incompressible heavy viscous fluid of variable density: An approximate theory. *Math. Proc. Cambridge* **51**, 179–201 (1955).
34. W. H. Reid, The effects of surface tension and viscosity on the stability of two superposed fluids. *Math. Proc. Cambridge* **57**, 415–425 (1961).
